# Are We Overtesting? Rethinking Routine Blood Work After Low-Risk Laparoscopic Cholecystectomy: A Retrospective Study

**DOI:** 10.3390/medicina61091555

**Published:** 2025-08-29

**Authors:** Murat Demir, Huseyin Kilavuz, Feyyaz Gungor, Sibel Yaman, Baki Ekci, Idris Kurtulus

**Affiliations:** Department of General Surgery, Basaksehir Cam and Sakura City Hospital, University of Health Sciences, Istanbul 34480, Turkey; drhuseyinkilavuz@gmail.com (H.K.); feyyaz.gngr@gmail.com (F.G.); syaman.ctf@hotmail.com (S.Y.); drbaki@yahoo.com (B.E.); idriskurtulus@gmail.com (I.K.)

**Keywords:** laparoscopic cholecystectomy, complication, postoperative blood tests

## Abstract

*Background and Objectives*: Laparoscopic cholecystectomy is one of the most performed surgical procedures worldwide. In low-risk patients, routine postoperative blood tests are frequently ordered despite limited evidence supporting their necessity. The aim of the study was to evaluate the predictability of complications that may occur with routine postoperative blood tests. *Materials and Methods*: This retrospective study examined 538 patients who underwent surgery in Cam and Sakura City Hospital between May 2020 and May 2021. Patients were divided into two groups: no postoperative complications (Group NC, n = 521) and postoperative complications (Group C, n = 17). Demographic characteristics, including age and gender, duration of surgery, cystic duct closure method, drain use, complications, preoperative and postoperative blood tests, and the mean time of hospital stay, were the collected data throughout the study. *Results*: The analysis of the post-operative blood test values revealed that the total bilirubin (*p* = 0.005), ALT (*p* = 0.002), AST (*p* = 0.002), GGT (*p* = 0.02) and amylase (*p* = 0.034) values were statistically significantly higher in Group C than in Group NC, but these values did not exceed the normal range except for ALT, AST and GGT, which were slightly higher than the normal parameters. Seventeen patients (3.15%) developed postoperative complications, including biliary leakage (n = 1); choledocholithiasis (n = 2); cardiac (n = 2), pulmonary (n = 9), and hemorrhagic (n = 2) complications; and a superficial wound infection (n = 1). Most complications were identified by symptoms and clinical observation. *Conclusions*: Routine postoperative blood tests in low-risk laparoscopic cholecystectomy patients do not significantly contribute to the early detection of complications. Clinical observation and targeted use of laboratory or imaging tests in selected high-risk cases might be more efficient. This approach can help reduce unnecessary workload, hospital costs, and healthcare expenditures without compromising patient safety.

## 1. Introduction

Gallstone disease is common, with a prevalence of 5–22% among the population [1]. Approximately three-quarters of patients are asymptomatic and one-quarter are symptomatic, and some of these patients may experience serious complications [2]. Symptomatic patients usually suffer from abdominal pain and dyspepsia. The first-choice treatment for symptomatic cholelithiasis is cholecystectomy. Approximately 1.5 million cholecystectomy surgeries are performed globally each year. Although the complication rate after these surgeries is low, it can be life-threatening when a complication occurs [3]. Many centers perform routine postoperative blood tests to detect complications early [4].

In surgical practice, it is common for individual general surgeons or surgical teams to rely on postoperative blood tests to help detect early complications after laparoscopic cholecystectomy. These tests are often perceived as useful tools to support clinical assessment and provide reassurance in the immediate postoperative period. However, these blood tests have high costs for both patients and insurance providers. Our clinical observations and experience have shown that in many cases (especially those with an uncomplicated surgical procedure), routine postoperative blood tests rarely provide additional information for the detection of complications. This observation has led us to question the necessity and diagnostic value of these tests over the detection of post-surgical complications. Therefore, we designed this study to evaluate the effectiveness of routine postoperative blood tests in detecting complications after laparoscopic cholecystectomy. This study aims to determine whether these tests provide significant clinical benefits or can be safely omitted in selected low-risk patients.

## 2. Materials and Method

Ethical approval for this study was obtained from the Ethics Committee of Cam and Sakura City Hospital (Approval Code: 2021/223, Approval Date: 13 October 2021). The study was conducted in accordance with the principles outlined in the Helsinki Declaration.

Retrospective data of patients who underwent cholecystectomy in Cam and Sakura City Hospital between May 2020 and May 2021 were evaluated. Patient data were retrieved from the institutional electronic medical record system. Data collection and curation were performed by MD, FG, and SY, while validation was independently conducted by MD and SY. Statistical analyses were performed by HK and FG, and software/database management was supervised by SY. The inclusion criteria were patients over the age of 18 who underwent elective laparoscopic cholecystectomy, whereas emergency operations, procedures converted to open surgery, patients who underwent endoscopic retrograde cholangiopancreatography (ERCP) or percutaneous cholecystostomy in the preoperative period, chronic cholecystitis related to recurrent biliary colic, a history of previous episodes of acute cholecystitis, patients with a history of previous abdominal surgery, patients with incomplete data, and patients with an American Society of Anesthesiologists (ASA) score of III and above in the preoperative evaluation were the exclusion criteria.

Patients were divided into subgroups based on postoperative complication onset (Group C: Patients with postoperative complications, Group NC: Patients with no postoperative complications). Demographic characteristics, including age and gender, duration of surgery, cystic duct closure method, drain use, complications, preoperative and postoperative blood tests, and the mean time of hospital stay were collected throughout the study.

All patients’ postoperative blood tests were performed approximately 22–24 h after surgery. The parameters of both preoperative and postoperative blood tests included white blood cell (WBC), C-reactive protein (CRP), total and direct bilirubin, alanine amino transferase (ALT), aspartate aminotransferase (AST), alkaline phosphatase (ALP), gamma-glutamyl transferase (GGT), amylase and lipase values.

### 2.1. Surgical Technique

All procedures were performed laparoscopically in the American position using the standard four-port technique (10 mm umbilical for camera, 10 mm epigastric for working hand, and two 5 mm ports in the right midclavicular and anterior axillary lines). Pneumoperitoneum was established with CO_2_ at 10–12 mmHg. Calot’s triangle was dissected with monopolar electrocautery. The cystic duct and artery were clipped (10 mm titanium; Large/Extra-large polymer clips were used for wide ducts) and divided. The gallbladder was removed in a retrieval bag via the epigastric port. Fascial closure was performed for 10 mm sites, and skin was closed with absorbable sutures or staples. Drain placement was at the surgeon’s discretion. All patients received perioperative antibiotic prophylaxis with a single dose of first-generation cephalosporin (1 g IV).

### 2.2. Biostatistical Data Analysis

The statistical analysis was performed using SPSS version 21 (IBM, New York, NY, USA). Descriptive statistics, including minimum, maximum, mean and standard deviation, were calculated. The normality distributions of the groups were evaluated with the Kolmogorov–Smirnov Test. Since the variables between the groups did not show a normal distribution, the non-parametric Mann–Whitney U test was applied. Categorical measurements were given as numbers and percentages, and continuous measurements were given as median, first quartile (Q1) and third quartile (Q3) values. The chi-square test was used for comparisons of proportions. For statistical significance, *p* values < 0.05 were considered significant.

## 3. Results

**Figure 1:**
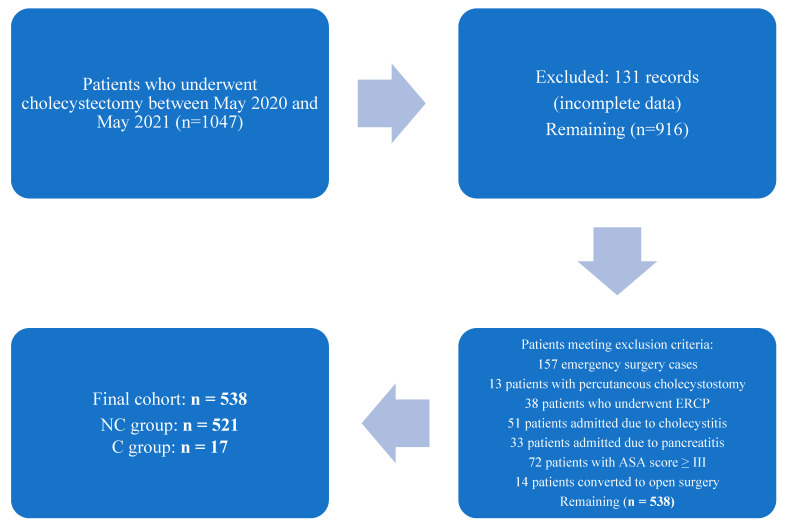
Flowchart illustrating the patient data selection and sampling process according to STROBE guidelines.

The initial database included 1047 patients who underwent cholecystectomy during the study period. After exclusion of 131 incomplete records and 157 emergency surgery cases, 759 patients were assessed for eligibility. Subsequently, 13 patients with percutaneous cholecystostomy, 38 patients who underwent ERCP, 51 admitted with cholecystitis, 33 with pancreatitis, and 72 with an ASA score ≥ III were excluded. Fourteen patients who were converted to open surgery were also excluded. The final study cohort consisted of 538 patients, including 521 in the NC group and 17 in the C group. A detailed flow chart according to STROBE guidelines is provided in Figure 1.

Among these, 521 patients (98.85%) in the NC group, and 17 patients (100%) in the C group had gallstones, and from the NC group, 1 patient (0.2–19%) had a gallbladder polyp and 5 patients (0.96%) had both gallstones and a gallbladder polyp. The mean age of the patients was 45.1 years (range: 18–85 years). There were 379 female patients (70.4%) and 159 male patients (29.6%). All procedures were performed under general anesthesia using a standard four-port laparoscopic technique. There were 521 patients in Group NC (no postoperative complication group) and 17 patients in Group C (postoperative complication group). The mean operation time was 58.4 (48–69) minutes. There were no intraoperative complications in any of the patients, and no mortality was observed. No conversion to open surgery was made. No patient required early re-operation. The mean length of hospital stay was 1.41 days in the NC group, whereas it was 6.41 days in the C group (*p* = 0.0009) (Table 1 and Table 2).

Preoperative blood tests revealed that the AST value in Group C was found to be significantly higher compared to the NC group, despite being within the normal range (*p* = 0.003). There were no significant differences among the remaining preop blood test parameters between both groups (*p* > 0.05) (Table 2).

During laparoscopic cholecystectomy, the cystic duct was closed using different methods depending on intraoperative conditions. The choice of closure method was made at the discretion of the operating surgeon, based on anatomical factors, tissue quality, and intraoperative safety considerations. In most patients (n = 517), endoclips were used for cystic duct ligation. Hemoclips were preferred in 20 patients, typically in cases where thicker or inflamed tissue was encountered. In one patient, a surgical stapler was preferred because of concerns regarding secure closure. Drainage was only performed in 301 patients (57.77%) in Group NC and in 10 patients (58.82%) in Group C. The decision to place a drain was based on the surgeon’s intraoperative judgment, considering factors such as bile leakage risk, bleeding, and local inflammation (Table 3).

The intraoperative findings of patients are shown in Table 4. Accordingly, a floppy gallbladder was encountered in 487 patients of the NC group, whereas 11 patients had a floppy gallbladder in group C, which was the most common intraoperative finding in both groups. The least common intraoperative findings were a buried gallbladder (n = 1) and an impacted stone (n = 1) in Group C and adhesion (n = 8) and an impacted stone (n = 6) in Group NC (Table 4).

The analysis of the post-operative blood test values revealed that the total bilirubin (*p* = 0.005), ALT (*p* = 0.002), AST (*p* = 0.002), GGT (*p* = 0.02) and amylase (*p* = 0.034) values were statistically significantly higher in Group C than Group NC, but these values did not exceed the normal range except for ALT, AST and GGT, which were slightly higher than the normal parameters (Table 4).

Postoperative complications occurred in 17 (3.15%) patients. These included wound infection (n = 1), bile leakage (n = 1), choledocholithiasis (n = 2), cardiac complications (n = 2), pulmonary complications (n = 9) and postoperative bleeding (n = 2). No blood product replacement was required for postoperative bleeding patients. ERCP was performed on one patient with bile leakage and two patients with choledocholithiasis. Other complications were managed conservatively or with minimally invasive interventions (Table 4).

## 4. Discussion

Complications are undesirable but possible outcomes following surgical procedures. Laparoscopic cholecystectomy, commonly performed for gallbladder diseases, is considered a safe and widely practiced surgical technique worldwide. The overall complication rate is approximately 1–4%, with most complications occurring within the first few days after surgery [5,6]. Bile duct injury, postoperative bleeding and biloma are the most common but still rare complications after laparoscopic cholecystectomy. Although they are rare complications, these can become life-threatening if not detected early [7,8]. Moreover, delayed recognition of postoperative complications—particularly after laparoscopic cholecystectomy—can lead to medicolegal consequences [9]. Therefore, early diagnosis and rapid management of complications are important to prevent clinical deterioration.

Previous studies have aimed to identify preoperative and intraoperative risk factors in patients undergoing laparoscopic cholecystectomy to define patient groups at higher risk for complications. Demographic and clinical characteristics such as advanced age, male sex, obesity (body mass index > 30 kg/m^2^), history of diabetes mellitus, and previous abdominal surgery; patients presenting with acute cholecystitis, especially those admitted emergently or requiring surgery more than 72 h after symptom onset, fever at admission (body temperature > 37.5 °C), chronic cholecystitis related to recurrent biliary colic, and a history of previous episodes of acute cholecystitis; and patients who have undergone prior endoscopic retrograde cholangiopancreatography (ERCP) are preoperative risk factors, and those patients with these factors are associated with increased surgical difficulty [10,11,12]. On the other hand, intraoperatively determined risks were found to be associated with a higher rate of complications in patients. These situations encountered during surgery can significantly increase the technical difficulty and risk of complications such as bile duct injury, bleeding, or conversion to open surgery. Anatomic difficulty, particularly the inability to clearly identify the cystic duct or safely dissect the Calot’s triangle; gallbladder gangrene; abscess formation; the presence of a buried gallbladder; the presence of impacted gallstones larger than 1 cm, particularly those lodged in the gallbladder infundibulum or Hartmann’s pouch; and the presence of bile or pus outside the gallbladder cavity and fistula are intraoperative risk factors [12,13,14,15]. Intraoperative findings in our study revealed that adhesion, anatomic difficulties, a buried gallbladder and impacted stones larger than 1 cm were the most common intraoperative risk factors which were encountered more commonly in group C, consistent with the abovementioned findings.

Identifying preoperative risk factors before surgery is essential for planning and optimizing patient care. Our study excluded patients with known high-risk factors and focused specifically on a low-risk patient population. This low-risk group was clearly defined and supported by favorable intraoperative findings. The primary objective of this study is to evaluate the necessity of routine postoperative blood tests in the low-risk patient group. We aim to demonstrate that routine laboratory tests may be unnecessary in this patient group, as they rarely contribute to the early detection of complications. Furthermore, these tests can increase healthcare costs for both patients and insurance providers without offering significant clinical benefits.

Early and timely intervention to the complications, especially after gallbladder surgery, usually yields satisfactory results [7,8,16]. To minimize the risk of missed complications, general surgeons and surgical teams commonly rely on routine blood tests and imaging studies, particularly ultrasonography (US) and routine abdominal drainage, as part of their daily postoperative care [4,17,18,19,20]. There are studies in the literature reporting that a temporary increase in liver function tests may be expected in the early period following laparoscopic abdominal surgery [21,22].

In determining the type and location of postoperative complications, which decrease as the surgeon’s experience increases, the correct and appropriate use of radiological imaging tests in the early and late periods may allow timely intervention. USG, CT, ERCP, MR, MRCP and radionuclide imaging all have a role in the evaluation of the postoperative patient [23,24]. Many studies have reported that clinical symptoms have higher specificity and sensitivity than biochemical parameters in detecting postoperative complications [4,25,26]. In line with this, some studies argue that routine blood tests after surgery are unnecessary, taking these conditions into account [22,25,26]. The objective of this study was to determine whether routine postoperative blood testing is truly necessary after laparoscopic cholecystectomy in clinically stable patients without prior abdominal or biliary surgery, no signs of acute cholecystitis, and those classified as ASA II or lower. Clarifying this could help eliminate redundant practices, reduce healthcare costs, optimize insurance expenditures, and streamline clinical workflow without compromising patient safety.

In patients with a long cystic duct, retained stones following cholecystectomy may lead to recurrent choledocholithiasis, leading to symptomatic biliary obstruction. Factors such as incomplete gallbladder resection, inadequate visualization of the gallbladder fossa, dense adhesions, active inflammation, excessive intraoperative bleeding, or an atypical gallbladder anatomy can contribute to stone retention in the cystic duct [27,28,29]. These conditions may manifest after laparoscopic cholecystectomy with symptoms of mechanical jaundice, including abdominal pain and fever [30].

Bile leakage is a known complication of laparoscopic cholecystectomy. Closure of the cystic duct is essential to prevent bile leakage, resulting in increased morbidity and the need for further intervention. Although leakage from the cystic stump is often classified as a minor complication, it can lead to serious outcomes, including intra-abdominal collections, sepsis, prolonged hospitalization, and, in rare cases, mortality. Early recognition of bile leakage is critical to minimize adverse outcomes [31,32]. The initial diagnosis is typically made using ultrasound or computed tomography (CT), with magnetic resonance cholangiopancreatography (MRCP) offering more detailed imaging of the biliary tree. In selected cases, endoscopic ultrasound (EUS) has also been used for diagnosis. Management options include surgical intervention or endoscopic retrograde cholangiopancreatography (ERCP), depending on the clinical scenario and anatomical considerations [30,33,34].

Although laparoscopic cholecystectomy (LC) is considered a safe and commonly performed procedure, postoperative bleeding remains a serious complication. Hemorrhage may occur intraoperatively or postoperatively, and if not recognized promptly, can lead to hemodynamic instability, reoperation, or even mortality. Injury to the cystic artery, liver parenchyma or abdominal wall vessels, anatomical variations of the biliary tree, a thickened gallbladder wall, gallbladder atrophy, a history of upper abdominal surgery, and multiple gallstones are causes of bleeding complications. Clinical manifestations can include abdominal distention, hypotension, tachycardia, and a drop in hemoglobin levels. Imaging studies or diagnostic re-laparoscopy may be required to confirm the source [35,36,37].

Among the total of 538 patients included in the study, 17 developed postoperative complications. Five of these cases were identified through careful clinical observation and close follow-up, underscoring the critical role of clinical vigilance in early diagnosis. Although routine blood tests were not the primary diagnostic tool in these patients, they provided useful supplementary information that supported clinical decision-making.

In our study, three patients developed biliary complications. Two were diagnosed with choledocholithiasis, and one experienced a postoperative bile leakage, which resulted in a prolonged hospital stay. All three patients were successfully treated with endoscopic retrograde cholangiopancreatography (ERCP) and discharged without further complications. Additionally, postoperative hemorrhage occurred in two patients. Bleeding was identified through close monitoring of the drain output and was observed to resolve spontaneously without the need for blood transfusion. Both patients were discharged uneventfully.

The pulmonary and cardiac complications observed in our study are consistent with those reported in clinical practice. Although laparoscopic cholecystectomy is associated with reduced postoperative pain, less atelectasis, and better preservation of pulmonary function compared to open surgery, pulmonary complications may still occur [38,39]. Factors such as abdominal wall or gallbladder bed inflammation, CO_2_ pneumoperitoneum, and patient positioning can contribute to complications like mild hypoxemia, basal atelectasis, or transient ventilation–perfusion mismatch [38,39,40,41].

Wound infection remains one of the most common postoperative complications and is influenced by surgical technique, patient comorbidities, and intraoperative factors [42]. Due to its minimally invasive nature, laparoscopic cholecystectomy generally carries a lower risk of infection. Although current guidelines recommend prophylactic antibiotics only for high-risk cases, routine administration remains common practice because intraoperative risk factors can be unpredictable [43,44]. In our study, all patients received 1 g (IV) of a first-generation cephalosporin as prophylaxis. Only one patient developed a superficial wound infection, which resolved with local wound care. The patient was discharged without further issues.

Strohäker et al. [25] showed that postoperative blood tests had low sensitivity. Similarly, Ben-Ishay et al. [26] reported that postoperative complications did not lead to a significant difference in blood tests. Although some studies argue that blood tests have high sensitivity in detecting infectious complications, they generally indicate that clinical symptoms are more important [4]. The results of our study indicated that routine postoperative blood tests were helpful in the clinical follow-up and overall assessment of these patients. However, these tests did not play a primary role in diagnosing pulmonary, cardiac, or wound-related complications.

One of the limitations of this study is the relatively small sample size, which may have limited the ability to detect a wider range of postoperative complications. Although a larger patient population could potentially reveal additional or rarer complications, the inclusion criteria and specific objective of the study were intentionally designed to focus on a well-defined, low-risk patient group. Despite this limitation, the findings obtained from the current sample are consistent with contemporary surgical practice and provide clinically meaningful insights that may inform future clinical decision-making.

In conclusion, routine postoperative blood tests in clinically stable low-risk patients undergoing laparoscopic cholecystectomy—those without prior abdominal or biliary surgery, no signs of acute cholecystitis, and classified as ASA II or lower—are neither necessary nor sufficient for detecting complications. Although some biochemical parameters were found to be higher in patients who developed complications, these values remained within normal limits. This suggests that routine blood tests are not reliable predictors of postoperative complications in this low-risk group. We believe that close clinical monitoring in this low-risk patient group is more effective in identifying early signs of complications. Selective use of laboratory and imaging tests in high-risk patients may help reduce unnecessary time, effort, hospital costs, and insurance expenditures.

## Figures and Tables

**Table 1:** General data and demographics.

Patient	n = 538
Age (yr) *	45.1 (18–85)
Gender **	
● Female	379 (70.4%)
● Male	159 (29.6%)
Duration of surgery (min) *	58.4 (48–69)
Postoperative complications **	17 (3.15%)

* (mean (min–max)); ** (n (%)).

**Table 2:** Distribution of age, etiology, hospital stay, and pre-op blood test parameters among groups.

Variables	Group NC (n = 521)	Group C (n = 17)	*p* Value
Age (yr) *	45.4 (18–85)	47.1 (39–54)	*p* = 0.616
Etiology **			*p* = 0.663
Gallbladder stones	515 (98.85%)	17 (100%)	
Gallbladder polyps	1 (0.19%)	0 (0)	
Gallbladder stones and polyps	5 (0.96%)	0 (0)	
Length of hospital stay *	1.41 (1–4)	6.41 (2–10)	***p* = 0.0009**
Pre-op blood test parameters *			
WBC (4.49–12.68 × 10^9^/L)	7.38 (6.1–8.56)	8.02 (6.73–9.6)	*p* = 0.94
CRP (0.5–5 mg/L)	1.7 (1–4.1)	3.4 (1.15–4.6)	*p* = 0.329
T. bilirubin (0–1.2 mg/dL)	0.39 (0.26–0.59)	0.42 (0.33–0.61)	*p* = 0.182
D. bilirubin (0–0.3 mg/dL)	0.15 (0.11–0.22)	0.15 (0.07–0.21)	*p* = 0.731
ALT (0–35 U/L)	17 (12.5–26)	25 (15–35)	*p* = 0.062
AST (10–35 U/L)	17 (14–21.5)	24 (18–31)	***p*** **= 0.003**
ALP (35–104 U/L)	68 (55–84)	63 (49–85)	*p* = 0.495
GGT (5–36 U/L)	21 (14–35)	18 (14–38.5)	*p* = 0.982
Amylase (28–100 U/L)	57 (47–71)	55 (35.5–78.5)	*p* = 0.525
Lipase (13–60 U/L)	29 (22–40)	37 (29.5–46)	*p* = 0.068

WBC; White Blood Count. CRP; C reactive protein. ALT; Alanine aminotransferase. AST; Aspartate Aminotransferase. ALP; Alkaline Phosphatase. GGT; Gamma-glutamyl transferase. * (mean (min–max)). ** (n (%)).

**Table 3:** Intraoperative variables.

Variables	Group NC (n = 521)	Group C (n = 17)
Cystic duct ligation type *		
● Endoclip	500 (95.97%)	17 (100%)
● Hemoclip	20 (3.84%)	0 (0)
● Stapler	1 (0.19%)	0 (0)
Drain *		
● Yes	301 (57.77%)	10 (58.82%)
● No	220 (42.23%)	7 (41.18%)
Intraoperative findings		
Floppy gallbladder	487	11
Adhesion (mild or high)	8	2
Anatomic difficulty **	11	2
Buried gallbladder	9	1
Impacted stone > 1 cm	6	1

* (n (%)), ** Difficult identification of the cystic duct, inadequate dissection of Calot triangle and inability to reach the critical view of safety.

**Table 4:** Post-operative blood test parameters and detailed rate and type of complications.

Variables	Group NC * (n = 521)	Group C * (n = 17)	*p* Value
Post-op blood test parameters *			
WBC (4.49–12.68 × 10^9^/L)	10.15 (8.44–12.36)	10.35 (8.5–12.33)	*p* = 0.966
CRP (0.5–5 mg/L)	6.7 (3.5–12)	11.3 (4.62–18.7)	*p* = 0.171
T. bilirubin (0–1.2 mg/dL)	0.46 (0.31–0.66)	0.7 (0.48–0.93)	***p* = 0.005**
D. bilirubin (0–0.3 mg/dL)	0.18 (0.14–0.27)	0.28 (0.14–0.46)	*p* = 0.062
ALT (0–35 U/L)	34 (23–53)	61 (33.25–148.5)	***p* = 0.002**
AST (10–35 U/L)	33 (24–46)	54 (33.25–147.5)	***p* = 0.002**
ALP (35–104 U/L)	65 (55–81)	72.5 (57.75–97)	*p* = 0.205
GGT (5–36 U/L)	24 (14–47)	62 (23–98)	***p* = 0.02**
Amylase (28–100 U/L)	48 (37–63)	65.5 (46.5–97.75)	***p* = 0.034**
Lipase (13–60 U/L)	23 (17–32)	28 (21.5–41.25)	*p* = 0.184
Overall complications **	n = 0	n = 17 (3.15%)	
Wound infection	0	1	
Bile leakage	0	1	
Choledocholithiasis	0	2	
Cardiac complications	0	2	
Pulmonary complications	0	9	
Hemorrhage	0	2	

WBC; White Blood Count. CRP; C reactive protein. ALT; Alanine aminotransferase. AST; Aspartate Aminotransferase. ALP; Alkaline Phosphatase. GGT; Gamma-glutamyl transferase. * (mean (min–max)); ** (n (%)).

## Data Availability

All data and materials are available and the corresponding author is Demir M.
